# The Node Deployment of Intelligent Sensor Networks Based on the Spatial Difference of Farmland Soil

**DOI:** 10.3390/s151128314

**Published:** 2015-11-11

**Authors:** Naisen Liu, Weixing Cao, Yan Zhu, Jingchao Zhang, Fangrong Pang, Jun Ni

**Affiliations:** 1 National Engineering and Technology Center for Agriculture/Jiangsu Key Laboratory for Information Agriculture/Collaborative Innovation Center for Modern Crop Production, Nanjing Agriculture University, Nanjing 210095, China; E-Mails: 2010201072@njau.edu.cn (N.L.); caow@njau.edu.cn (W.C.); yanzhu@njau.edu.cn (Y.Z.); pangfangrong@njau.edu.cn (F.P.); 2 Jiangsu Collaborative Innovation Center for the Technology and Application of Internet of Things, Nanjing 210095, China; 3 Jiangsu Key Laboratory for Eco-Agricultural Biotechnology around Hongze Lake/Collaborative Innovation Center of Regional Modern Agriculture & Environmental Protection, Huaiyin Normal University, Huai’an 223300, China; 4 Nanjing Institute of Agricultural Mechanization of National Ministry of Agriculture, Nanjing 210014, China; E-Mail: zhangjc9@163.com

**Keywords:** intelligent sensor network, deployment, farmland soil differences, coverage degree, cost, fuzzy c-means clustering, normalized intra-cluster coefficient of variation

## Abstract

Considering that agricultural production is characterized by vast areas, scattered fields and long crop growth cycles, intelligent wireless sensor networks (WSNs) are suitable for monitoring crop growth information. Cost and coverage are the most key indexes for WSN applications. The differences in crop conditions are influenced by the spatial distribution of soil nutrients. If the nutrients are distributed evenly, the crop conditions are expected to be approximately uniform with little difference; on the contrary, there will be great differences in crop conditions. In accordance with the differences in the spatial distribution of soil information in farmland, fuzzy c-means clustering was applied to divide the farmland into several areas, where the soil fertility of each area is nearly uniform. Then the crop growth information in the area could be monitored with complete coverage by deploying a sensor node there, which could greatly decrease the deployed sensor nodes. Moreover, in order to accurately judge the optimal cluster number of fuzzy c-means clustering, a discriminant function for Normalized Intra-Cluster Coefficient of Variation (NICCV) was established. The sensitivity analysis indicates that NICCV is insensitive to the fuzzy weighting exponent, but it shows a strong sensitivity to the number of clusters.

## 1. Introduction

The real-time monitoring and exact diagnosis of crop growth are instructive to control crop development and enhance crop yields. Traditionally, crop growth information has been obtained by field sampling and laboratory analysis. Although this approach can generate reliable results, it is costly and inefficient, which fails to meet the precise management modern crop production requirements for real-time, large-scale and long-term crop growth information. With the development of spectral analysis technology, the method of monitoring crop growth information by using the canopy reflectance spectra of crops has been widely studied [1,2,3,4,5,6,7]. On this basis, some sensors for crop conditions and portable monitoring instruments for crop growth have been developed [8,9,10,11,12], which has laid a foundation for obtaining crop growth information rapidly and in real-time. In recent years intelligent WSNs, as a new monitoring technology, have been utilized to collaboratively and comprehensively obtain the target information through a large number of sensors deployed in a target area and self-organized sensor networks. At present, this technique has been widely applied to various fields, including military [13], transportation [14], environmental monitoring [15], agriculture [16], *etc*. Due to the characteristics of agricultural production, namely vast areas, scattered fields and long growth cycle of crops, WSNs are able to meet the needs of precise agriculture for obtaining real-time farmland information in the temporal-spatial domain. For example, a WSN was used by Burrell *et al.* [17] to supervise the eco-environment parameters of a vineyard, such as the temperature, humidity, light and soil moisture to accurately harvest and control grape production and they established favorable application effects. Using WSN data, Xiao *et al.* [18] monitored the soil moisture content and the water thickness on the surface of soil for water-saving irrigation of rice. Srbinovska *et al.* [19] utilized a WSN to monitor the environmental information in a greenhouse to economically cultivate vegetables and reduce the administration costs. Moreover, a WSN was applied by Abd El-Kader to precisely cultivate potatoes to improve the yield and quality so as to achieve increased economic benefits [20].

The deployment model for sensor nodes, as an important part of WSN research, determines the quality of service of a WSN. In general, the more the deployed sensor nodes, the larger the information coverage, and the better the sensory ability of the network. However, excessive nodes often bring about high costs and redundancy of sensory information. Hence, the problem of how to minimize the number of sensor nodes on the premise that the information is still monitored completely needs to be solved first. At present, some scholars aim to evenly deploy the sensor nodes by using different methods, for example, regular grids, such as triangle, square, or regular hexagon shaped [21,22], glowworm swarm optimization algorithm [23], virtual force algorithm [24], *etc.* In these methods, the target zone will be perceived repeatedly by several sensors so that the information collected is too redundant. In order to reduce the network deployment costs, Chakrabarty *et al.* [25] deployed two kinds of nodes with different costs together based on the regular grid deployment mode. In short, this kind of deployment plays a role in saving costs, but it is not universal so that it is limited in actual application. Aitsaadi *et al.* [26] divided the target area into key and non-key monitoring areas to non-uniformly deploy the sensor nodes in a target area by using artificial potential fields and a tabu search heuristic algorithm, where the deployment density of nodes was connected with the probability of the key monitoring events in the target area. This method was appropriate for application scenarios like fire monitoring, while in crop production and management, the monitoring probability density of crop growth information throughout the farmland was similar, therefore, it failed to satisfy the demands of providing crop growth information in a wide-area farmland environment, namely full coverage and low costs.

In the process of crop production and management, under the same cultivation conditions, the crop condition differences are mainly influenced by the spatial distribution of soil nutrients. In other words, if the spatial distribution of soil nutrients is even, the differences in crop condition are slight; if not, the differences are great. When the crop condition in an area of farmland is uniform, the crop growth information at each point in this area can be adopted to stand for the growth trend of the whole area. In accordance with the differences in the spatial distribution of soil information in farmland, fuzzy c-means clustering was applied to divide the farmland into several areas, where the soil fertility of each area is nearly uniform. Then the crop growth information in the area could be monitored with complete coverage by deploying a sensor node there. The WSN deployed by this method was able to completely monitor the crop growth information under a wide-area farmland environment, while at the same time it could greatly decrease the number (and hence cost) of deployed sensor nodes.

## 2. The CGMD302 Crop Growth Information Sensor

**Figure 1:**
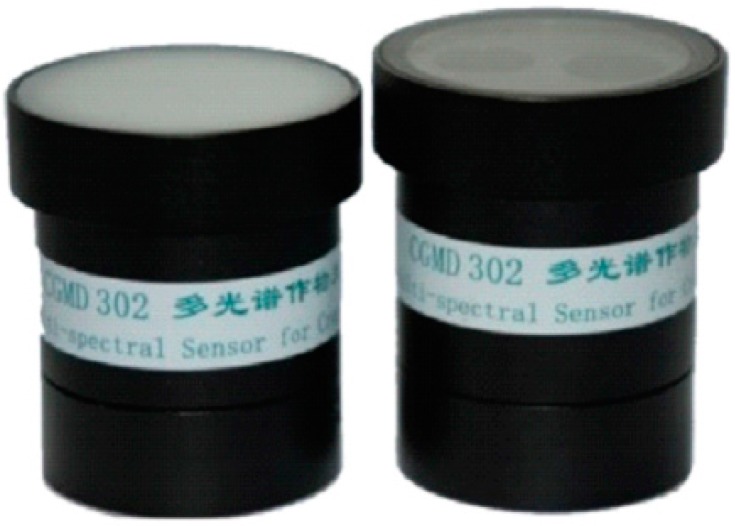
Multi-spectral sensor for crop growth.

CGMD302 crop growth information sensor is a kind of device for perceiving crop growth information based on canopy reflectance spectra. It was developed by National Engineering and Technology Center for Information Agriculture, Nanjing Agricultural University, China in the light of the research achievements on the sensitive wavebands of rice and wheat growth indexes for several years. The sensor was made up of two kinds of detection lenses (720 nm and 810 nm), which were applied to measure the spectral reflectance of crop canopy characteristics. Besides, it was utilized for coupling the spectrum monitoring model for growth indexes to provide crop growth information, including the leaf nitrogen content, leaf nitrogen accumulation, leaf area index, leaf dry weight, *etc.* In the sensor system, sunlight was used as the light source and a light filter was adopted for light splitting. As for the structure, it was divided into an upwelling light sensor and a downwelling light sensor, where the former was utilized to receive the radiation information of sunlight at 720 nm and 810 nm bands for cosine correction, while the latter was employed to receive the light radiation information of the crop canopy reflection at the corresponding bands. The structure parameters of detection lens are as follows: diaphragm aperture of 12.8 mm, hole depth of 26 mm and field angle of 27°; and the performance parameters are: Spectral filter bandwidth of 10 nm with transmittance of 65%~70%. The sensitivity and spectral responsivity of the chosen photoelectric detector are 0.55 A/W and 0.011 A/(w/cm^2^) respectively. With the parameter matching, each detection lens was composed of spectral filters and photoelectric detectors. It was characterized by simple light path, high reliability of signal transmission and it was also convenient to be integrated and transplanted. The sensor broke through the disadvantages of traditional sensors for growth information, such as the complex light path and use of a large number of optical devices. Furthermore, it was packaged by using a cylindrical aluminium shell with 38 mm and 50 mm in aperture and height, respectively. Owing to its light weight and small volume, the sensor was very applicable in fields (see Figure 1).

## 3. Fuzzy C-means Clustering

Fuzzy c-means (FCM) clustering is a common unsupervised clustering algorithm that works by maximizing the similarity of the observed values divided into the same cluster and minimizing the similarity of the different clusters. In this algorithm, the membership of each sample point to all clustering centers is obtained by optimizing the objective function to determine the cluster of sample points so as to achieve the purpose of automatically clustering sample data. Assuming that data set X is performed fuzzy c-means clustering:

(1) $$X=\left[\begin{array}{ccc} x_{11} & \ldots & x_{1f}\\ \vdots & \vdots & \vdots \\ x_{n1} & \ldots & x_{nf} \end{array}\right]=\left(x_{ab}\right)$$

where f is the number of clustering factors, n is the number of samples, $x_{ab}$ is the numerical value of the *b^th^* factor in the *a^th^* sample, (a=1,⋯,n, b=1,⋯,f), $x_{a}=\left(x_{a1},\cdots ,x_{ab}\right)$ is the *a^th^* sample in data set X.

Data set X is divided into *c* clusters, $C=\left\{C_{1},\cdots ,C_{c}\right\}$, 1<c<n. The clustering center $V=\left\{{\text{v}}_{1},\cdots ,{\text{v}}_{c}\right\}\left({\text{v}}_{i}=\left(v_{i}^{1},\cdots ,v_{i}^{n}\right)\right)$, where c is the number of clusters, and $v_{i}^{f}$ is the clustering center of the *i^th^* cluster of factor *f*. The membership matrix $U=[u_{ik}]$, $u_{ik}\in [0,1]\left(i=1,\cdots ,c;k=1,\cdots ,n\right)$, where c is the number of clusters, n is the number of samples, and $u_{ik}$ stands for the membership of the *k^th^* sample belonging to the *i^th^* cluster, and it meets $\sum_{i=1}^{c}u_{ik}=1\;\left(k=1,2,\cdots ,n\right)$. In fuzzy c-means clustering algorithm, the clustering analysis is conducted by iterating the minimum objective function, where the common objective function is:

(2) $$J_{m}(U,V)=\sum_{k=1}^{n}\sum_{i=1}^{c}{\left(u_{ik}\right)}^{m}{\left(d_{ik}\right)}^{2}$$

where n is the number of samples, c is the number of clusters, *m* is the fuzzy weighted exponent (1≤m<∞), *U* is the membership matrix, and V is the clustering center. During clustering based on soil data, the values of m can vary from 1.2 to 1.5 [27]. ${\left(d_{ik}\right)}^{2}={\left\|x_{k}-v_{i}\right\|}^{2}$, where $d_{ik}$ represents the Euclidean distance between the *k^th^* sample and the *i^th^* clustering center.

Through iteration analysis, update the clustering center $v_{i}$ using Equation (3) and update the fuzzy cluster matrix $u_{ik}$ using Equation (4). When the change of the clustering centers before and after iteration is less than the preset threshold value, or the specified times of iterations are achieved, the iteration process is terminated.

(3) $$v_{ik}=\frac{\sum_{k=1}^{n}{\left(u_{ik}\right)}^{m}x_{k}}{\sum_{k=1}^{n}{\left(u_{ik}\right)}^{m}},1\leq i<c$$

(4) $$u_{ik}={\left[\sum_{j=1}^{c}{\left(\frac{{d_{ik}}^{2}}{{d_{jk}}^{2}}\right)}^{m}\right]}^{-1}$$

## 4. Cluster Validity

The optimal cluster number of fuzzy c-means clustering is determined by the discriminant function, among which fuzziness performance index (FPI) and normalized classification entropy are frequently used [28]. The membership matrix *U* is employed in the computation process of FPI and NCE, where the line of the matrix means the membership for all data points in the sample pertaining to the cluster, and the column refers to the membership of a data point in each cluster, and the sum of the membership in each column is 1. If a membership degree in a column is close to 1, while the other membership degrees are approximately 0, then it suggests that the cluster at the data point is clear and the clustering effect is favorable. On the contrary, if all membership degrees are close, *i.e.*, the membership is 1/c, it is difficult to determine the cluster of data points and the clustering effects are poor:

(5) $$FPI=1-\frac{cF-1}{c-1}$$

(6) $$F=\frac{1}{n}\sum_{k=1}^{n}\sum_{i=1}^{c}{\left(u_{ik}\right)}^{2}$$

where $u_{ik}$ is the membership, n is the number of samples, c is the number of clusters, 1/c≤F≤1. *F* represents the overlap degree of data among different clusters. With the increase of c, F shows a monotonic decrease [29]. FPI is the normalized form of F, where 0≤FPI≤1. When FPI is 0, the inter-cluster data are non-overlapping, *i.e.*, no data are shared, which is called hard clustering; while when FPI is 1, the inter-cluster data are totally shared so that it is unable to cluster effectively. Hence, the smaller the FPI, the more effective the clustering results.

NCE can be calculated by Equation (7):

(7) $$NCE=\frac{nH}{n-c}$$

(8) $$H=-\frac{1}{n}\sum_{k=1}^{n}\sum_{i=1}^{c}u_{ik}\log_{a}(u_{ik})$$

where $u_{ik}$ is the membership, n is the number of samples, c is the number of clusters and *H* is the entropy function [30]. The lower the degree of sharing of the inter-cluster data, the more distinct the clustering and the better the clustering effects, so the smaller the H. During hard clustering, the sharing degree of data is 0, which means that *H* reaches the minimum, 0. On the contrary, when the data between different clusters are completely shared, it fails to cluster and the clustering effect is the worst. At that time, the membership is 1/c, and H is maximized, namely $\log_{a}c$. The variation trend of N​CE is consistent with that of H [31], $NCE\in [0,n\log_{a}c/(n-c)]$. Thereby, the smaller the NCE, the better the clustering effects.

These methods utilizing the process information of fuzzy c-means clustering, such as FPI and NCE, are called internal standard methods [32], in contrast, the methods without using the data during clustering analysis are referred to as external standard methods [32]. The inherent defect of the former is that sometimes they fail to effectively determine the optimal clustering of data sets [33,34], FPI and NCE included. In this case, the other methods are needed to determine the optimal cluster number [35]. When both FPI and NCE are minimized, the corresponding cluster number is selected as the optimal cluster number [28,34]. As a general rule, these two methods are employed simultaneously, but both FPI and NCE are sensitive to the fuzzy weighted exponent *m*. It is reported that the optimal cluster number determined by FPI and NCE changes with the changing *m* [36]. In addition, *m* is a parameter determined subjectively during fuzzy c-means clustering, that is to say, *m* is with uncertainty. Due to its sensitivity to *m*, the reliability of determining the optimal cluster number by FPI and NCE is reduced. Based on the idea of establishing the external standard method, the variation of intra-cluster and inter-cluster data after fuzzy c-means clustering was applied to construct the normalized intra-cluster coefficient of variance (NICCV) of discriminant function to determine the optimal cluster number of fuzzy c-means clustering. The discriminant function is operated on the basis of the internal difference law of data after clustering and it is insensitive to the fuzzy weighted exponent *m*. Therefore, it can effectively determine the optimal cluster number of fuzzy c-means clustering.

### 4.1. Normalized Intra-Cluster Coefficient of Variance

The data set in Equation (1) was subjected to fuzzy c-means clustering, where the reasonable clustering results were with the minimal differences in the intra-cluster data and the maximal differences in the inter-cluster data. The differences between data were denoted by coefficient of variation in this paper. In order to reduce the influence of sample capacity, the inter-cluster data variation was expressed as average of inter-cluster coefficient of variation (AICCV), as shown in Equation (9):

(9) $$AICCV=\frac{1}{(c-1)\cdot f}\cdot \sum_{i=1}^{c}\sum_{j=1}^{f}\frac{CV_{ij}}{n_{ij}}$$

where c is the number of clusters, f is the number of clustering factors, $n_{ij}$ is the number of samples for the factor *j* in the *i*th cluster, $CV_{ij}$ is the coefficient of variation of the samples for the factor *j* in the *i*th cluster. The coefficient of variation can be computed by Equation (10):

(10) $$CV=\frac{S}{\bar{x}}$$

where $\bar{x}$ stands for the arithmetic mean of samples, and S is the standard deviation of sample.

The external clusters coefficient of variation (*ECCV*) is calculated by using the clustering centers of each factor in all clusters after clustering (see Equation (11)):

(11) $$ECCV=\frac{1}{f}\sum_{k=1}^{f}CV_{k}$$

where $CV_{k}$ is the coefficient of variation of the clustering centers the factor *k* in each cluster after clustering, and f is the number of clustering factors. The NICCV can be obtained by normalizing AICCV (see Equation (12)):

(12) $$NICCV=\frac{AICCV}{ECCV}=\frac{\sum_{i=1}^{c}\sum_{j=1}^{f}\frac{CV_{ij}}{n_{ij}}}{(c-1)\cdot \sum_{k=1}^{f}CV_{k}}$$

The smaller the value of NICCV, the better the clustering effects. The cluster number corresponding to the minimum value of NICCV is considered as the optimal cluster number. Hence, the corresponding clustering results are the most reasonable.

### 4.2. Verification

In this work, reasonably dividing farmland is the key technique for WSN deployment, so the accuracy of the optimal classification number determined by discriminant functions is essential when using a fuzzy c-means clustering algorithm to divide the farmland. The properties of discriminant functions can be tested by data sets. The data sets used need to be endowed with distinct classification numbers, so that whether the classification number determined by discriminant functions is correct or not can be judged. This research chose six data sets to verify the properties of NICCV, among which, five of them were made artificially. To more accurately test the properties of NICCV, the spatial distribution of the data sets was provided with complexity. The 6th data set is Iris, which is a well-known machine learning data set and has been frequently used in the validation of discriminant functions [37,38].

**Figure 2:**
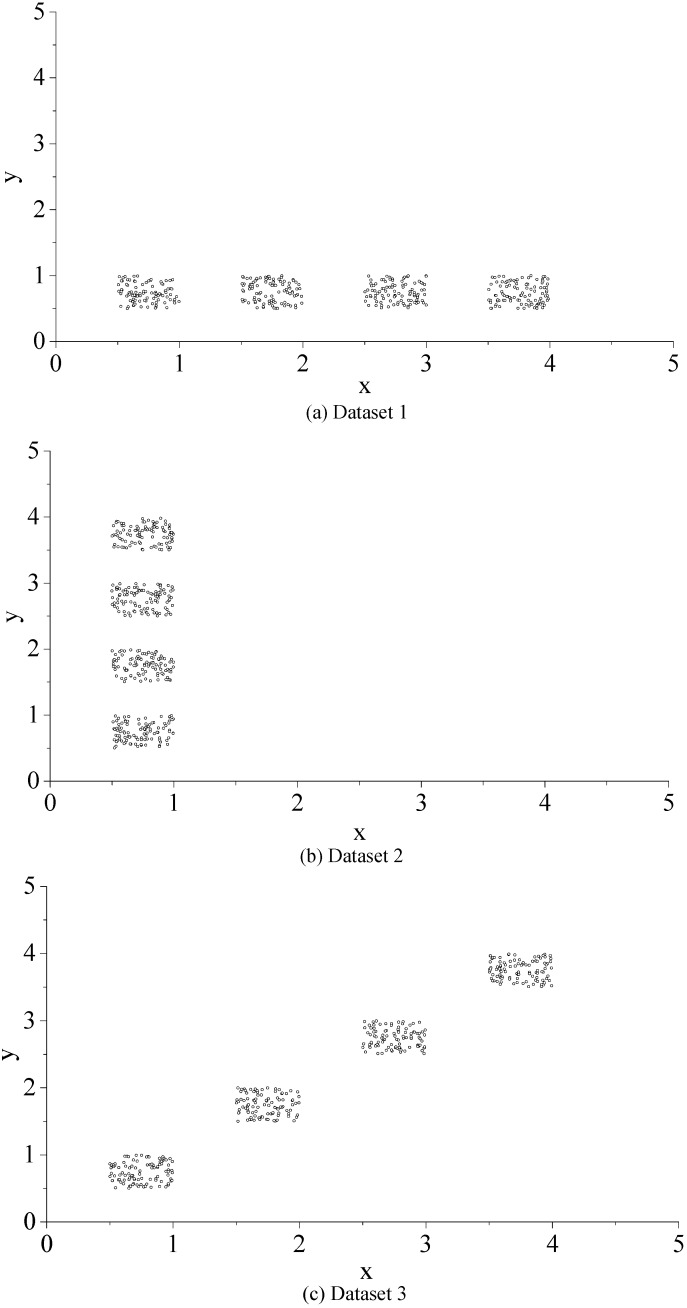

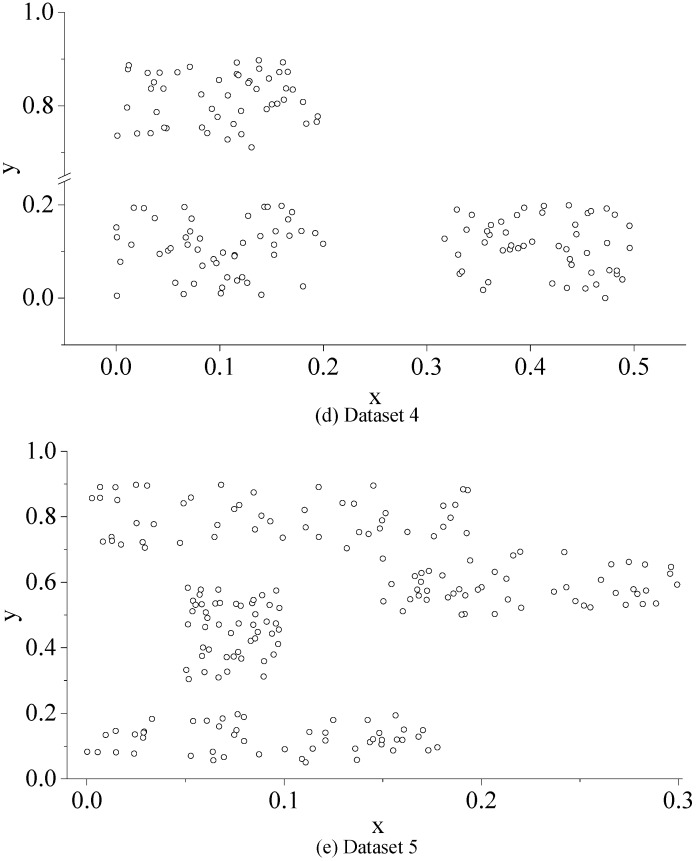
Data sets for experiment. (**a**) Dataset 1; (**b**) Dataset 2; (**c**) Dataset 3; (**d**) Dataset 4; (**e**) Dataset 5.

Data Sets 1–3 are geared to two-dimensional data, and their distributions are indicated in Figure 2a–c. As shown in these figures, all three of these data sets can be divided into four clusters. The values of the four clusters of data in Data Set 1 are in the same range in the *y*-dimension, while the value ranges in the *x*-dimension are different and non-overlapping. On the contrary, the value ranges of the data in Data Set 2 in the *x*-dimension are the same, but different and non-overlapping in the *y*-dimension. The four clusters of data in Data Set 3 show a ladder-like distribution, which suggests that the values in both *x*-dimension and *y*-dimension are various and without overlap. The value ranges of Data Sets 1 and 2 overlap only in the *x*-dimension or *y*-dimension, while the values of Data Set 3 are non-overlapping in both the *x*-dimension and *y*-dimension. It explains that the data in these three data sets are spatially distributed in a simple way. Data Sets 4 and 5 can be clustered as three and four clusters, respectively (see Figure 2d,e). There are overlaps in the value ranges for the three clusters of data in Data Set 4 and the four clusters of data in Data Set 5, which indicates that the spatial distributions of the data are relatively complex. Thereby, it is more difficult to determine the optimal cluster number for the discriminant function.

The Iris data set belongs to the UCI database proposed by the University of California Irvine (Irvine, CA, USA) for machine learning. There are now 187 data sets in the database. Initially, the Iris data set was data for geographic variation extracted from Iris flowers on the Gaspar Peninsula, which was first proposed by Anderson. The data set contains 150 samples, which are pertinent to three subgenera of *Iris L*, namely *Iris setosa* Pall. ex Link, *Iris versicolor* L., and *Iris virginica* L. Four characteristics are employed for the quantitative analysis of samples, including the length and width of calyx and petal. The data structure of the Iris data set is complex, so it can verify the performance of discriminant functions.

Experiment 1 aimed to test the performance of discriminant functions NICCV, FPI and NCE in determining the optimal cluster number by using Data Sets 1–3, while through Data Sets 4 and 5, the performance of discriminant function was examined in Experiment 2. Besides, Experiment 3 verified the performance of the discriminant function with the Iris data set.

#### 4.2.1. Experiment 1

**Figure 3:**
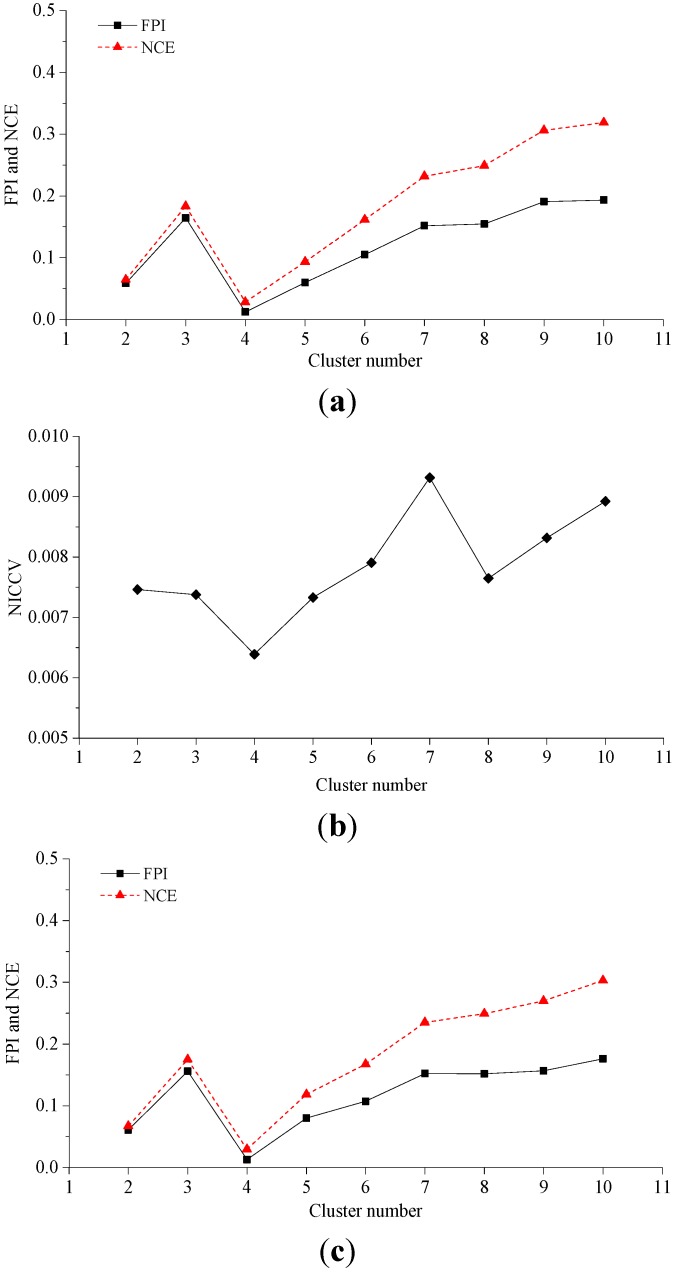

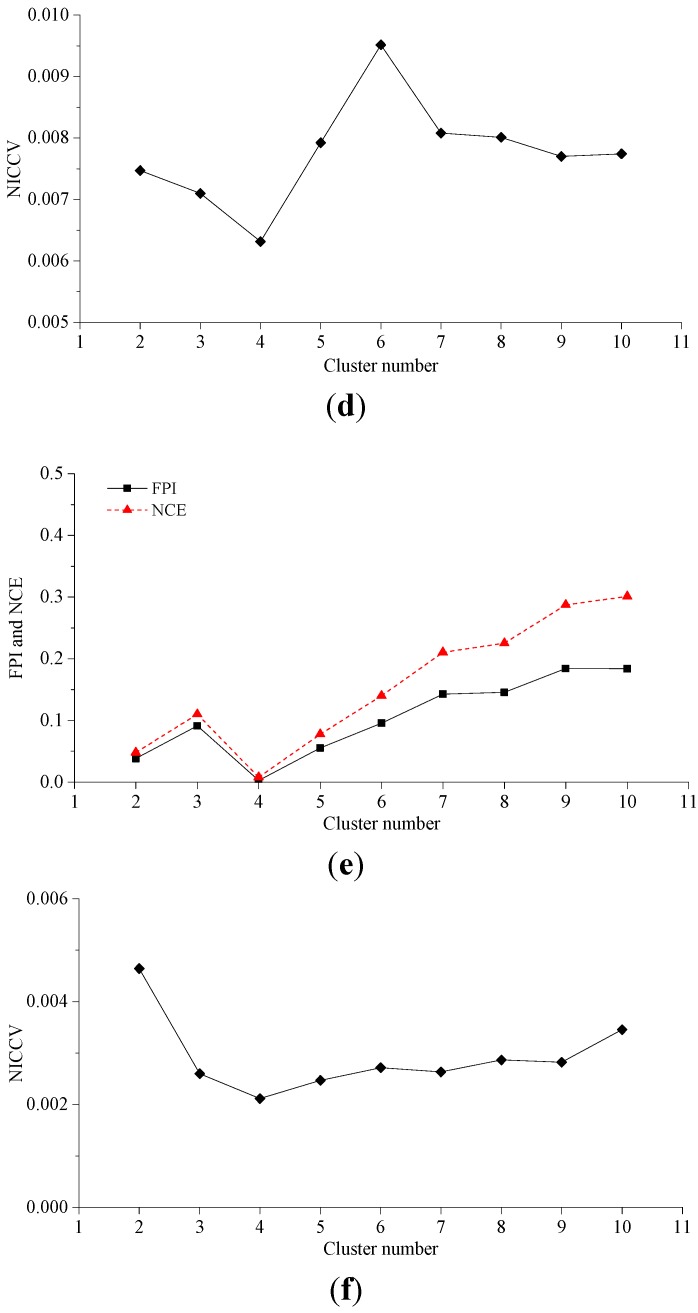
Dataset 1–3 clustering analysis. (**a**) Corresponding FPI and NCE to the different cluster numbers of Dataset 1; (**b**) Corresponding NICCV to the different cluster numbers of Data Set 1; (**c**) Corresponding FPI and NCE to the different cluster numbers of Dataset 2; (**d**) Corresponding NICCV to the different cluster numbers of Dataset 2; (**e**) Corresponding FPI and NCE to the different cluster numbers of Dataset 3; (**f**) Corresponding NICCV to the different cluster numbers of Dataset 3.

The fuzzy c-means clustering algorithm was adopted for the clustering of Data Sets 1–3. The fuzzy weighted exponent *m* was set as 1.5 to calculate NICCV, FPI and NCE after clustering (see Figure 3). Figure 3a illustrates the corresponding FPI and NCE to the different numbers of clusters after clustering Data Set 1. When the number of clusters is four, FPI and NCE reach the minimum. This means that the optimal cluster number determined by FPI and NCE is four. In addition, Figure 3b indicates the corresponding NICCV of the different numbers of clusters after the clustering of Data Set 1. When the number of clusters is four, NICCV is minimized, *i.e.*, the optimal cluster number determined by NICCV is four. Meanwhile, Data Set 1 can be clustered into four categories. It is demonstrated that FPI, NCE and NICCV all provide the correct cluster number. Both Data Sets 2 and 3 can be clustered into four categories. The corresponding FPI and NCE to the different cluster numbers of Data Sets 2 and 3 after clustering are shown in Figure 3c,e, respectively, while the corresponding NICCV of both data sets are illustrated in Figure 3d,f. It is demonstrated that the cluster numbers provided by FPI, NCE and NICCV are proper. Experiment 1 shows that FPI, NCE and NICCV provide the accurate number of clusters for the data sets with simple spatial distribution.

#### 4.2.2. Experiment 2

**Figure 4:**
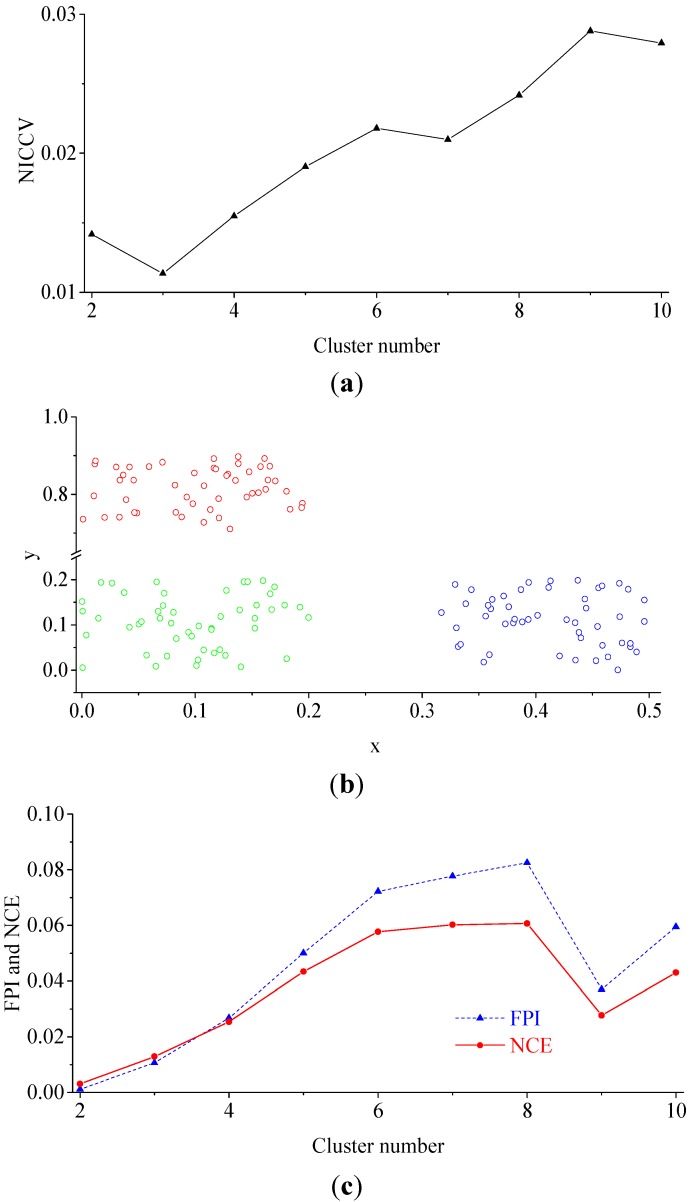

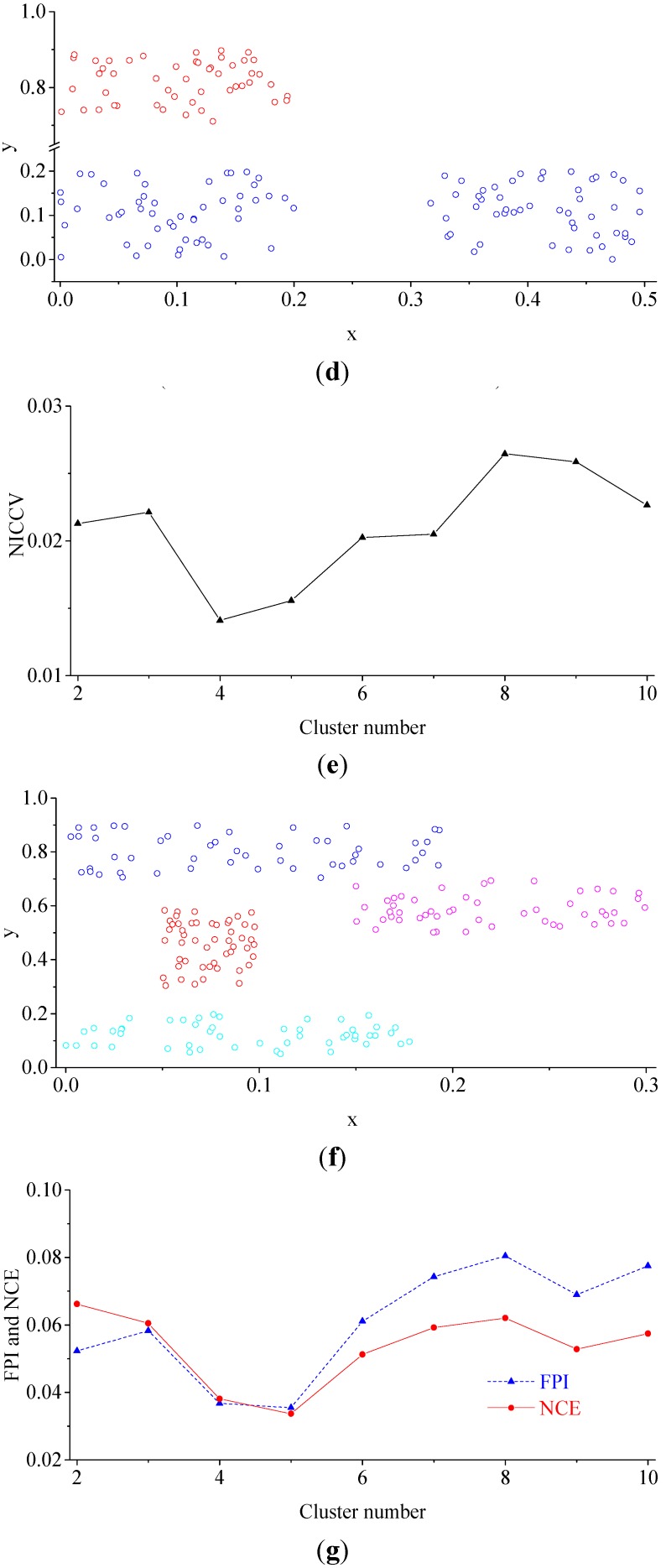

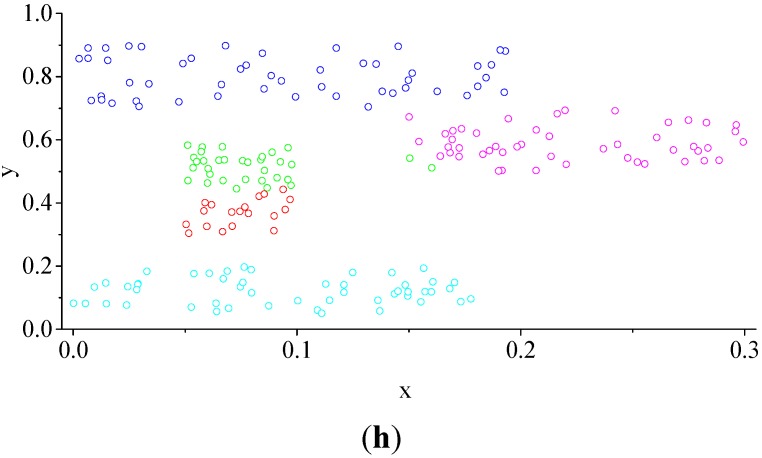
Dataset 4–5 clustering analysis. (**a**) Corresponding NICCV to the different cluster numbers of Dataset 4; (**b**) Cluster number of Dataset 4 determined by NICCV is 3 (the same cluster shown with the same color); (**c**) Corresponding FPI and NCE to the different cluster numbers of Dataset 4; (**d**) Cluster number of Dataset 4 determined by FPI and NCE is 2 (the same cluster shown with the same color); (**e**) Corresponding NICCV to the different cluster numbers of Dataset 5; (**f**) Cluster number of Dataset 5 determined by NICCV is 4 (the same cluster shown with the same color); (**g**) Corresponding FPI and NCE to the different cluster numbers of Dataset 5; (**h**) Cluster number of Dataset 5 determined by FPI and NCE is 5 (the same cluster shown with the same color).

Data Sets 4 and 5 contain artificially constructed data, which can be clustered into three and four categories, respectively. These two data sets are clustered by using fuzzy c-means clustering, where the parameter *m* is selected as 1.5. After clustering, the values of discriminant functions, including FPI, NCE and NICCV are calculated. The changes in the values of discriminant functions with the changing cluster numbers and the clustering results of Data Sets 4 and 5 are shown in Figure 4. Figure 4a indicates that the optimal cluster number of Data Set 4 determined by NICCV is three, and the clustering result is correct (see in Figure 4b). As illustrated in Figure 4c, the optimal cluster number of Data Set 4 determined by FPI and NCE is two, but the clustering result is incorrect (see Figure 4d). It can be seen from Figure 4e,g that the optimal cluster numbers of Data Set 5 determined by NICCV as well as FPI and NCE are four and five, respectively. Among them the cluster number determined by the former is proper, but that by the latter is wrong. The corresponding clustering results are shown in Figure 4f,h.

#### 4.2.3. Experiment 3

**Figure 5:**
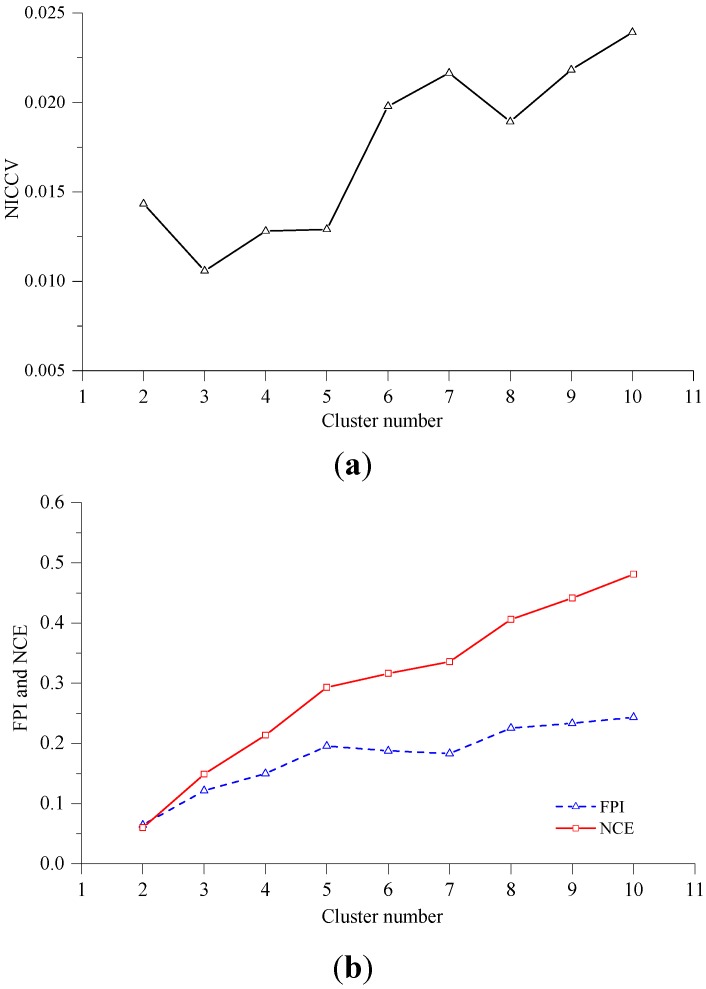
NICCV, FPI and NCE under different clustering results. (**a**) Corresponding NICCV to the different cluster numbers of Iris Data set, the cluster number of Iris Data set determined by NICCV is 3; (**b**) Corresponding FPI and NCE to the different cluster numbers of Iris Data set, the cluster number of Iris Data set determined by FPI and NCE is 3.

In this experiment, the Iris standard test data set was employed as clustering data. The Iris data set is often utilized to test the validity of discriminant functions [37,38], of which the data are more complex than those of Data sets 4 and 5. The fuzzy c means clustering was applied for the clustering analysis of the data set, where *m* = 1.5. The FPI, NCE and NICCV under different clustering results are indicated in Figure 5. Figure 5 shows that the optimal cluster number provided by NICCV is three, and the result is appropriate, while the data set is falsely clustered into two categories by FPI and NCE. Michael found that the optimal cluster number provided by discriminant functions F and *H* are inaccurate during the clustering the Iris data set [37,38], where both F and *H* belong to internal standard methods. The discovery by Michael also validated that there are defects of the internal standard methods in practical application.

As shown by Experiments 1–3, FPI and NCE provide the correct cluster number for the three data sets in Experiment 1, but the optimal cluster numbers identified for the data sets in Experiment 2 and Iris data set in Experiment 3 are wrong. The situation may be connected with the complexity of the spatial distribution for the data in different data sets. There are overlaps in the value ranges of the data set used by Experiment 2 in both the *x*-dimension and *y*-dimension, while the values of Data sets 1 and 2 in Experiment 1 overlap in the *x*-dimension or *y*-dimension, and the value range of Data set 3 is non-overlapping in both the *x*-dimension and *y*-dimension. Obviously, the spatial distribution of the data sets in Experiment 2 is more complicated than that of the data sets in Experiment 1. Moreover, the Iris data set is 4-dimensional, for which the complexity of the multi-dimensional data distribution is far greater than that of Data sets 1 and 2. Comparing with FPI and NCE, NICCV provides the proper cluster numbers for all data sets with various complexities utilized in Experiments 1–3. It is demonstrated that NICCV can preferably recognize the data with different complexities in spatial distribution and the performance of the optimal cluster number determined by NICCV is superior to that of FPI and NCE.

#### 4.2.4. The Sensitivity of Discriminant Functions to *m*

**Figure 6:**
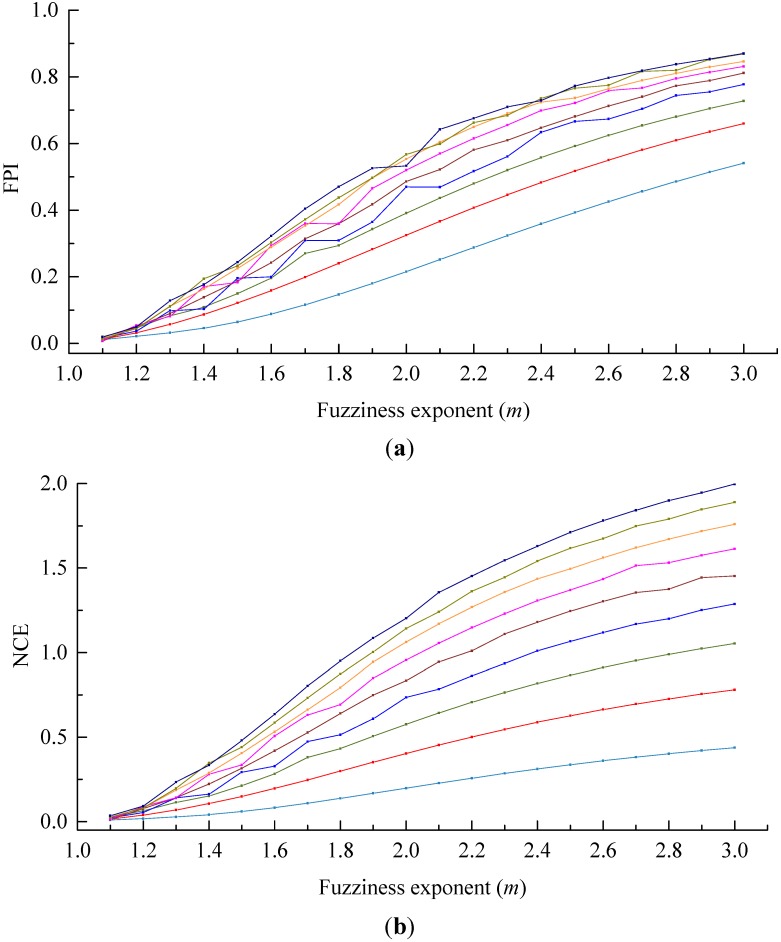

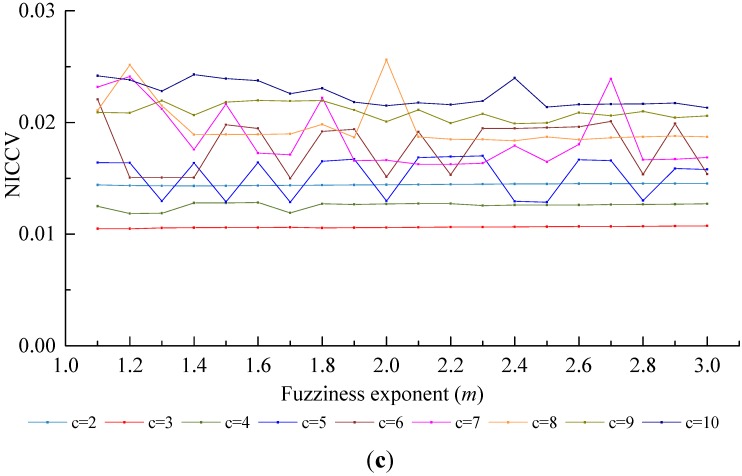
Sensitivity analysis of FPI and NCE, NICCV to *m*. (**a**) Corresponding FPI to the different *m*; (**b**) Corresponding NCE to the different *m*; (**c**) Corresponding NICCV to the different *m*.

The discriminant functions are employed to determine the optimal cluster number for clustering, so they should be insensitive to the parameters in the clustering process [37], such as fuzzy weighted exponent *m*. Roubens found that with the increase of the number of clusters c, both FPI and NCE monotonously increased in case of the membership was invariable [31]. Windham discovered that both FPI and NCE were sensitive to the number of clusters c and the fuzzy weighted exponent *m*, besides, both of them failed to effectively provide the correct cluster number [37]. During clustering the soil attribute data, some scholars also found that FPI is sensitive to the fuzzy weighted exponent *m* [27,29]. The fuzzy weighted exponent *m* is one of the clustering process parameters, which is determined artificially, *i.e.*, *m* is uncertain. In consequence, the sensitivity of discriminant functions to *m* will influence the validity of the decided optimal cluster number. In this paper, the Iris data set was applied to analyze the sensitivity of FPI and NCE, NICCV to *m* (see Figure 6). Figure 6 indicates that FPI and NCE are sensitive to *m*. As *m* increases, both FPI and NCE show an increasing trend. Moreover, when *m* increases from 1.2 to 2, FPI and NCE raise greatly and slow down later. If *m* is preset, in general, FPI and NCE increase with the increase of the number of clusters c, which manifests that there are interactions between *m* and *c*, while when *m* increases, NICCV is still a horizontal line or fluctuates slightly, *i.e.*, the overall trend is horizontal. It suggests that NICCV is insensitive to *m*. In addition, when c=3, the values of NICCV are the minimal and be a horizontal line as *m* changes. Thus, the optimal cluster number determined by NICCV for Iris data set is 3. Besides, when m varies, NCE always keeps minimum when the number of clusters is two, which suggests that the optimal cluster number determined by NEC for Iris data set is two. When 1.2≤m≤3.0, the optimal cluster number provided by FPI for Iris data set is two, while when m=1.1, the optimal number is six.

#### 4.2.5. The Sensitivity of Discriminant Functions to the Number of Clusters *c*

**Figure 7:**
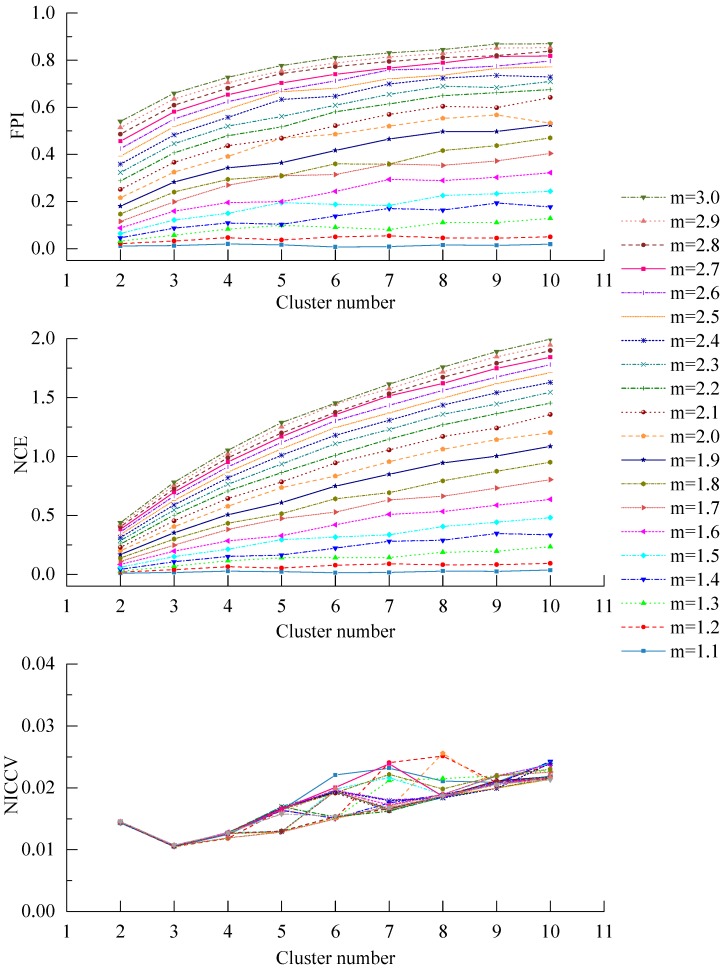
Sensitivity analysis of FPI and NCE, NICCV to *c*.

The sensitivity of FPI and NCE, NICCV to the number of clusters *c* was analyzed by using the Iris data set (see Figure 7). As presented in Figure 7, when *m* ranges from 1.1 to 1.5, FPI and NCE change slightly, which explains that they are insensitive to *c*. Within the scope of *m* from 1.6 to 3.0, FPI and NCE increase obviously with the increasing *c*, *i.e.*, they are sensitive to *c*. The amplitude of variation of NCE is much greater than that of FPI under the same *m*. As the number of clusters *c* increases, in general, NICCV increases linearly, but it reaches the minimum value when *c* is equal to the correct cluster number. It is demonstrated that NICCV is sensitive to *c*. Furthermore, owing to NICCV is insensitive to *m*, the NICCV with different *m* values changes with *c* intensively.

The test results of NICCV, FPI and NCE on six data sets revealed that NICCV is capable of determining correct classification number of test data sets. AS the data sets show simple spatial distribution as seen in Data Sets 1, 2 and 3, FPI and NCE can provide correct classification number; for the case that data sets have complex spatial distribution as Data Sets 4, 5, and Iris, the classification number determined was proved to be all wrong. The sensitivity analysis indicated that NICCV is not sensitive to the cluster process parameter *m* of fuzzy c-means clustering when determining optimal classification number, however it is found to be sensitive to FPI and NCE. This outcome indicated that NICCV is more suitable to determine the classification number of fuzzy c-means clustering. This work divided the farmland based on the soil properties to further deploy a WSN, and could use NICCV to determine the optimal classification number of the farmland.

## 5. Dividing the Farmland Based on the Spatial Difference of Soil Nutrients

The farmland was divided reasonably so as to make sure each divided subarea has same soil nutrients approximately. By doing so, the consistent growth conditions of crops in each subarea can be ensured. By deploying one node in subareas divided, the monitor for the information of the growth conditions of crops can be realized in the current node. The properties of soil influencing the growth of crops mainly include total nitrogen (TN) content, organic matter (OM) content, available phosphorus (AP) content, available potassium (AK) content and soil electrical conductivity (EC). Based on the soil nutrient conditions, this paper used FCM and NICCV to divide a farmland zone with an area of 5 hectares in Rugao City of Jiangsu Province, China. On this basis, the deployment of WSN nodes for monitoring crop growth conditions can be realized so as to acquire the information such as leaf nitrogen content, leaf nitrogen accumulation, leaf area index, leaf dry weight, *etc.* in the growth of crops.

**Figure 8:**
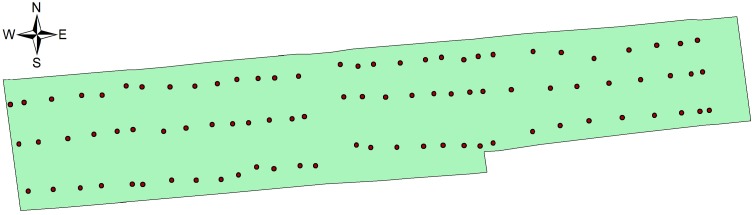
Soil sampling points and boundary of study area.

The center of the field is at 120°45′42.923′′ E and 32°16′4.425′′ N. A sampling point was selected at the both ends and middle of all natural fields in the research area, and the distance between the adjacent sampling points in the whole research area was less than 50 m. A EM38-MK2 ground conductivity meter, produced by Geonics Limited (Mississauga, Ontario, Canada), was utilized to randomly measure the conductivity in the range of 1 m around sampling points three times. Then the average was used as the electrical conductivity (EC) of the sampling point. Besides, three samples of topsoil (from 0 to 20 cm) in the same area was collected randomly at the same range and the topsoil was well mixed as the representative sample at that point. In total, 90 samples were collected. Figure 8 illustrates the distribution of the sampling points. The measuring items include: Organic matter (OM) content, total nitrogen (TN) content, the content of available phosphorus (AP) and available kalium (AK).

**Figure 9:**
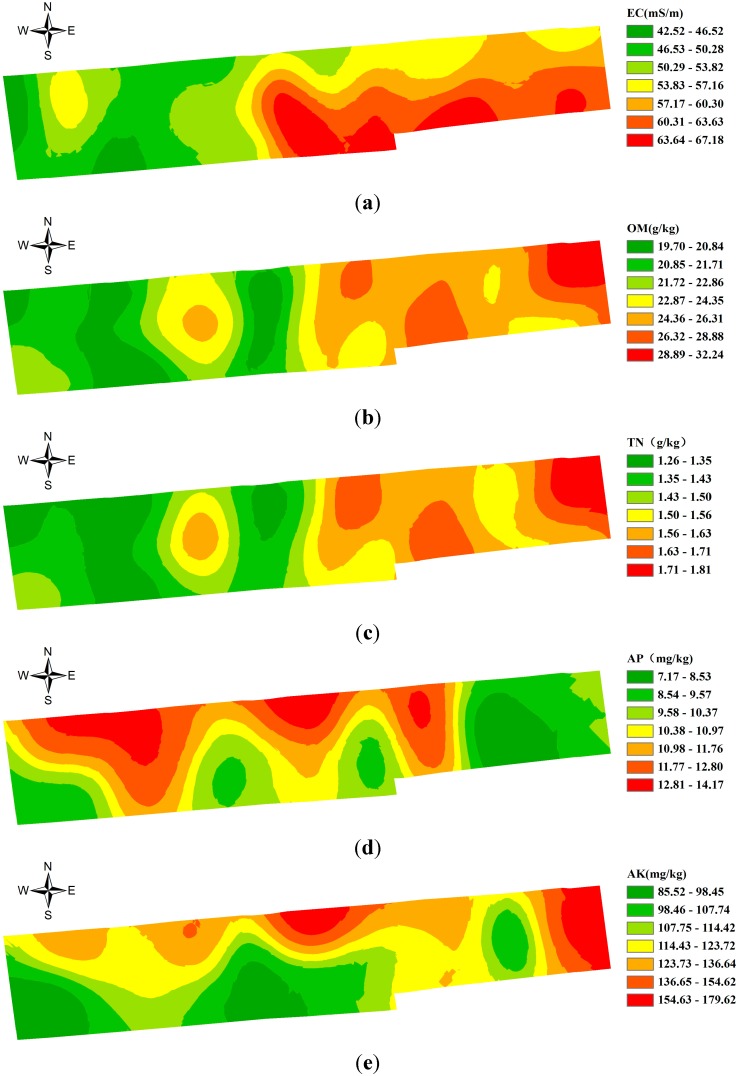
Spatial distribution maps of soil attributes in the farmland. (**a**) The spatial distribution of the EC of soil; (**b**)The spatial distribution of the OM content of soil; (**c**) The spatial distribution of the TN content of soil; (**d**) The spatial distribution of the AP content of soil; (**e**)The spatial distribution of the AK content of soil.

The semi-variance function model was applied to the optimum fitting of the soil attribute data in research area (Table 1). The results manifest that the structural variation of soil attributes led by spatial autocorrelation is greater than random variation on the field scale, where EC and AK show strong spatial correlations, OM, TN and AP have medium spatial correlations. Through the ordinary Kriging interpolation of the unsampled area, the area data of the research area were acquired to indicate the spatial distribution of the soil attributes in the farmland (see Figure 9).

**Table 1:** Fitting results of soil attribute data.

Soil Properties	Model	Semi-Variance Function Model Parameters					
		Nugget	Sill	Nugget/Sill (%)	Range (m)	*R*^2^	RSS
EC (mS/m)	Spherical	4.559	28.753	15.95	131.02	0.956	0.0157
OM (g/kg)	Spherical	0.0531	0.193	27.51	84.77	0.825	0.0038
TN (g/kg)	Spherical	1.52 × 10^−4^	3.31 × 10^−4^	45.92	91.23	0.869	0.0225
AP (mg/kg)	Exponential	3.85	7.701	49.99	149.7	0.908	0.0688
AK (mg/kg)	Exponential	4.50 × 10^−4^	0.072	0.63	65.2	0.873	0.0055

**Figure 10:**
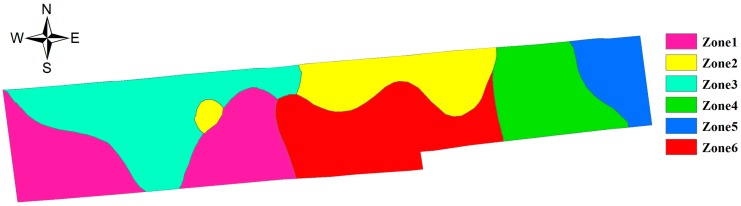
Optimal management zone map in the area.

Rugao farmland was divided by using a fuzzy c-means clustering algorithm, and the actual division was made based on soil attribute data, *i.e.*, electrical conductivity, organic matter, total nitrogen content, available phosphorus content and available potassium content. The fuzzy weighted exponent *m* was set to 1.5 and cluster number *c* was set from 2 to 15. Testing different data sets showed that the cluster number provided by NICCV was accurate, therefore NICCV was utilized to determine the optimal cluster number after the Rugao farmland was divided by the fuzzy c-means clustering algorithm. NICCV reaches its minimum value for cluster number 6, in other words, six is the optimal cluster number for the Rugao farmland. Eight fields were thus obtained (see Figure 10).

As shown in Figure 9 and Figure 10, the distribution of the contents in OM, TN and AK in zone 4 is shown to accord with that of Zone 5; while Zones 1–3,6 integrate the spatial distribution features of the contents in TN, OM, AP, AK and EC. The significance of different soil nutrient indexes between different subareas was compared at multiple levels by using Duncan’s new multiple range method (see Table 2). As indicated in Table 2, in general, the coefficients of variation of soil nutrients at each subarea decline to different extent. Moreover, the differences in each soil nutrient index between subareas are significant. It is noted that the difference of the subareas decreased and soil nutrients tended to be consistent. This was conducive for network deployment. By deploying sensor nodes in each subarea so as to further form a network, the information for the crop growth in each subarea could be monitored.

**Table 2:** Zoning statistics for coefficients of variation of soil properties.

Zones	EC		OM		TN		AP		AK	
	Mean mS·m^−1^	CV %	Mean g·kg^−1^	CV %	Mean g·kg^−1^	CV %	Mean mg·kg^−1^	CV %	Mean mg·kg^−1^	CV %
Zone 1	49.43d	9.59	21.54c	12.63	1.401c	12.60	9.46bc	13.60	94.74d	15.80
Zone 2	55.38c	4.42	25.28b	11.42	1.590b	11.08	12.67a	22.73	144.39b	26.34
Zone 3	50.55d	8.16	21.76c	8.26	1.387c	9.91	12.74a	11.59	127.58bc	22.95
Zone 4	61.54ab	4.28	24.39b	8.59	1.549b	8.20	8.12c	23.82	116.57cd	25.42
Zone 5	58.5bc	7.05	31.33a	11.38	1.800a	10.80	8.47c	20.43	173.17a	24.78
Zone 6	63.8a	6.55	24.73b	12.62	1.580b	10.67	10.52b	20.90	110.00cd	12.01
total	55.35	12.25	23.66	14.46	1.501	12.96	10.71	25.41	120.33	29.80

Different normal letters indicate significant difference at level of 0.05.

## 6. Comparing the Performance of Deployment Methods

The deployment method presented in this paper was compared with several deployment methods based on regular grids on two aspects, including area per node (APN) and the number of the deployed nodes. The regular grids involved included regular hexagon, square and equilateral triangle. The maximal APNs of the three regular grids were computed by using the method proposed by Bai *et al.* [21]:

(13) $$\gamma_{\max}^{Hex}=\frac{3}{4}\sqrt{3}{\left(\min\left\{R_{s},R_{c}\right\}\right)}^{2}$$

(14) $$\gamma_{\max}^{Squ}=2{\left(\min\left\{R_{s},\frac{\sqrt{2}}{2}R_{c}\right\}\right)}^{2}$$

(15) $$\gamma_{\max}^{Tri}=\frac{3}{2}\sqrt{3}{\left(\min\left\{R_{s},\frac{\sqrt{3}}{3}R_{c}\right\}\right)}^{2}$$

**Figure 11:**
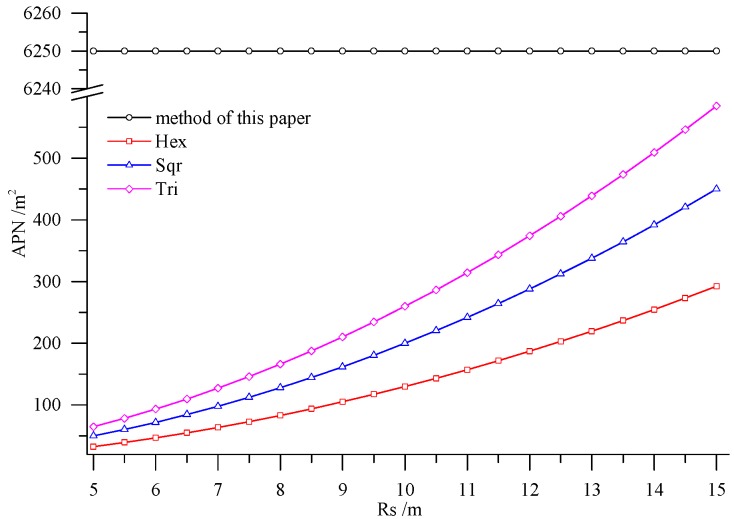
APN with different deployment methods.

**Figure 12:**
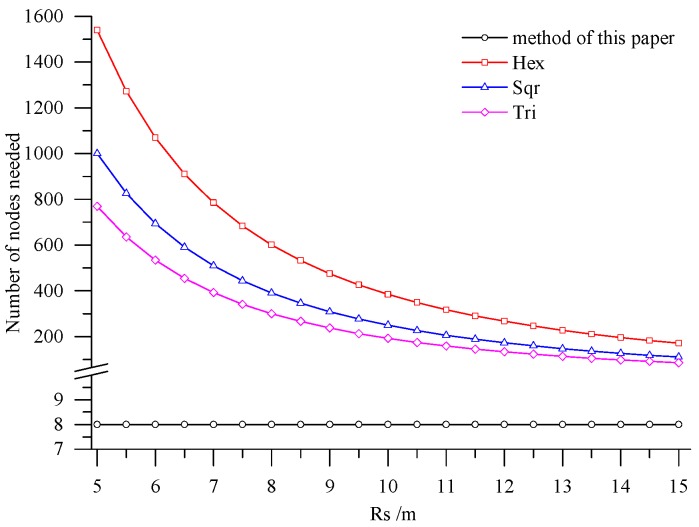
The number of nodes needed with different deployment methods.

$\gamma_{\max}^{Hex}$, $\gamma_{\max}^{Squ}$ and $\gamma_{\max}^{Tri}$ are respectively the maximal APNs of a regular hexagon, square and equilateral triangle, $R_{s}$ and $R_{c}$ are the perception radius of nodes and the signal transmission distance of nodes. In reality, most of the transmission distances of nodes are within the range of 200 m to 500 m, so the transmission distance of nodes $R_{c}$ was set as 200 m here. The transmission distance could guarantee the connectivity of the deployed nodes in Rugao farmland after division. The perception radius of nodes was related to the sensors employed. The research area in Rugao farmland covered about 5 hm^2^. There were eight nodes deployed by using the method proposed here with APN of 6250 m^2^. In addition, the APN did not change with the changing perception radius of nodes, but it was in connection with the spatial differences of farmland soil. In other words, if the difference was great, the APN was small, otherwise, the APN was large. Figure 11 shows that the APNs of different regular grids increase rapidly with the increase of the perception radius of nodes, while all of them are much less than the APN calculated by the method utilized in this paper. Figure 12 presents the number of the required nodes by the method in this paper and the deployment methods based on regular grids. As illustrated in Figure 12, the nodes required by the latter are much more than those of the former. The superiority of the method presented in this paper is clearly demonstrated.

## 7. Conclusions

The coverage and cost of intelligent WSNs are important factors influencing the deployment of sensor nodes. The growth conditions of crops are mainly affected by soil nutrients, *i.e.*, if the soil nutrients are similar, the crop conditions are nearly uniform. According to the spatial distribution of soil nutrients, the farmland was divided to ensure that the properties of each soil nutrient in farmland were similar. After that, a sensor node was deployed at each field to completely monitor the farmland information at a low cost:

1. In accordance with the farmland soil attribute data, including the organic matter content, total nitrogen content, available phosphorus content, available potassium content, electrical conductivity, *etc.*, fuzzy c-means clustering algorithm was utilized to divide the farmland. In order to accurately judge the optimal cluster number of fuzzy c-means clustering, a discriminant function for NICCV was established. NICCV was constructed on the basis of the variation of the intra-cluster and inter-cluster data after clustering. It was verified that NICCV could provide the correct cluster number for the test data with both simple and complex spatial distribution by using simulation data and the Iris standard test data set. Moreover, its performance was obviously superior to that of FPI and NCE. As indicated by the sensitivity analysis, FPI, NCE and NICCV were sensitive to the number of clusters c, *i.e.*, all of them increased with the increase of c. Besides, FPI and NCE showed a strong sensitivity to the fuzzy weighted exponent *m*, which suggests that both of them raised with the increasing m. Moreover, when *m* was different, they might provide various optimal cluster numbers. On the contrary, NICCV was insensitive to m. Thus, the NICCV with different cluster numbers still could be a horizontal line or fluctuate within a narrow range horizontally as m changed.
2. Combining with the crop growth characteristics and features of sampling the crop growth information in farmland, the low-cost sensor node deployment was achieved based on the premise of completely monitoring the crop growth information. Compared with existing methods, the perception radius of sensor nodes did not need to be considered in this method. Through comparing the sensor node deployment for a farmland with an area of 5 hm^2^, it can be known that the APN of the method presented in this paper was 6250 m^2^, and only eight nodes were applied. However, when the perception radius of nodes was 15 m, the APNs of the deployed sensor networks based on the three kinds of regular grids, namely regular hexagon, square and equilateral triangle, were from 250 m^2^ to 600 m^2^, which suggests that 200–300 nodes were needed. In practical application, the perception radius of the sensors for crop growth information is relatively small. If the perception radius is 5 m, the APNs of the network deployed by the three regular grids are less than 100 m^2^, so that the number of required nodes is up to 800–1600, which means that deploying the sensor network nodes costs a lot. By comparison, it is demonstrated that the node deployment method in this paper is preferable in applications, which can guarantee the complete information monitoring and also minimize the node deployment costs.
3. Crop growth is mainly influenced by soil nutrients; in addition, soil moisture and the NDVI of previous crop also have an important impact on crop growth. Information about soil moisture and NDVI can be obtained through sensors, NDVI can also be acquired through satellite remote sensing. If there is a large variation in the spatial distribution of soil moisture, it needs to be considered as one of the factors influencing farmland division. The ability of maintain soil water is given by its type, e.g., clay has a great ability to retain water, while the ability of sand is very limited. For this reason, soil type may substitute soil moisture as one of the criteria in dividing farmland. Different crops need different amounts of water and nutrients, as well as different times in the growing season. Furthermore, cropping system and crop phenology also affect crop growth. For this reason, our future work will consider all these factors in order to improve network deployment algorithm, so it can adapt to more complex application of wireless sensor network.
